# Extracellular Vesicles in Cardiovascular Diseases: Diagnosis and Therapy

**DOI:** 10.3389/fcell.2022.875376

**Published:** 2022-06-01

**Authors:** Xiaojing Zhang, Yuping Wu, Qifa Cheng, Liyang Bai, Shuqiang Huang, Jun Gao

**Affiliations:** ^1^ Department of Pharmacy, The Sixth Affiliated Hospital of Guangzhou Medical University, Qingyuan People’s Hospital, Qingyuan, China; ^2^ Department of Scientific Research, The Sixth Affiliated Hospital of Guangzhou Medical University, Qingyuan People’s Hospital, Qingyuan, China; ^3^ Department of Clinical Medicine, The Third Clinical School of Guangzhou Medical University, Guangzhou, China; ^4^ Department of Clinical Medicine, The Sixth Clinical School of Guangzhou Medical University, Guangzhou, China

**Keywords:** EVS, cardiovascular diseases, diagnosis, prognosis, therapies

## Abstract

Cardiovascular diseases (CVDs) are the leading cause of global mortality. Therapy of CVDs is still a great challenge since many advanced therapies have been developed. Multiple cell types produce nano-sized extracellular vesicles (EVs), including cardiovascular system-related cells and stem cells. Compelling evidence reveals that EVs are associated with the pathophysiological processes of CVDs. Recently researches focus on the clinical transformation in EVs-based diagnosis, prognosis, therapies, and drug delivery systems. In this review, we firstly discuss the current knowledge about the biophysical properties and biological components of EVs. Secondly, we will focus on the functions of EVs on CVDs, and outline the latest advances of EVs as prognostic and diagnostic biomarkers, and therapeutic agents. Finally, we will introduce the specific application of EVs as a novel drug delivery system and its application in CVDs therapy. Specific attention will be paid to summarize the perspectives, challenges, and applications on EVs’ clinical and industrial transformation.

## Introduction

Cardiovascular diseases (CVDs) are the highest rate of death around the world (Balakumar et al., 2016). The mortality of atherosclerotic cardiovascular disease (ASCVD) including ischaemic heart disease (IHD) and ischaemic stroke is as high as 40% in China (Zhao et al., 2019). It accounts for 17.3 million deaths globally per year and is expected to account for >23.6 million deaths per year by 2030 (Mozaffarian et al., 2015). CVDs have become a major health issue affecting global economic and social development. Currently, cardiovascular drug innovation meets major challenges, including widely varying outcomes, and persistent CVD treatment costs rise, improvement of “upstream factors” such as social status, self-empowerment, education, and health revenue (McClellan et al., 2019).

Over the past decade, although traditional pharmacotherapy and surgery can alleviate the symptoms of CVDs and reduce the mortality rate (Roth et al., 2017; Zelniker and Braunwald 2020), there is still lack of clinical strategy for repairing damaged myocardium after myocardial infarction (MI) or preventing the catastrophic development of heart failure (HF) (Andersson and Vasan 2018). Traditional medication is less invasive, but it can cause organs damage, or other serious side effects (Lassiter et al., 2020). Despite the excellent effect, the clinical application of cardiac surgery is always limited by the complex procedures and postoperative complications (Roth et al., 2020). The prognosis of CVDs remains poor. Therefore, new strategies and methods are urgently needed for CVDs therapy.

With the application of the human genome project and molecular biology, targeted therapies (macromolecular drugs, gene editing technologies, nucleic acid drugs, and cell therapy) were applied to the CVDs (Xu and Song 2021), especially since cell-based therapy in CVDs has been intensively studied worldwide. Numerous preclinical studies showed that cell-based therapeutic strategies have emerged as the most promising option for CVDs through repairing and replacing the damaged vascular and cardiac tissues, then improving cardiac function (Afzal et al., 2015; Wollert et al., 2017; Xu et al., 2021). However, challenges include the insufficient number of implanted stem/progenitor cells, the poor survival rate of transplanted cells in the ischaemic cardiac tissue, the impaired reparative ability of stem/progenitor cells in patients with CVDs, predisposition to cardiac arrhythmias, cardiac hypertrophy, and cancer limit the clinical efficacy of cell-based therapy (Passier et al., 2008; Chen et al., 2021; Xu et al., 2021).

Recently, with the continuous research on extracellular vesicles (EVs), the roles of EVs in CVDs have been gradually recognized. Therefore, systematic research on EVs is necessary for the clinical diagnosis, prognosis, and therapy development in CVDs (Chong et al., 2019; Han et al., 2021). EVs exist in blood, urine, saliva, amniotic fluid, malignant ascites, breast milk, and so on (Jansen et al., 2017), which are nano-sized, enclosed by a lipid bilayer, and secreted by virtually all cell types, including exosomes, microvesicles (MVs) and apoptotic bodies (Fu et al., 2020). EVs can carry proteins, lipids, messenger ribonucleic acids (mRNAs), micro-ribonucleic acids (miRNAs), and deoxyribonucleic acids (DNAs). Compared with other biological carriers, EVs exhibit the function of transmitting information between cells in biological processes such as inflammation, blood coagulation, vascular regulation, cell proliferation, and apoptosis (de Abreu et al., 2020; Sanwlani and Gangoda 2021). EVs can be used as clinical markers for coagulation function, inflammatory response, and tissue as well as organ damage diagnosis (Berumen Sánchez et al., 2021). EVs may act as a clinical therapeutic agent for regulating vascular homeostasis, correcting coagulation, improving the internal environment, and protecting tissue function (Colombo et al., 2014). Interestingly, accumulating evidence showed that CVDs cause vascular endothelial cells and cardiomyocytes damage. Then EVs were released into the extracellular environment and participated in the process of CVDs. In addition, EVs are involved in many physiological and pathological development of CVDs, including angiogenesis (Beltrami et al., 2017), cardiomyocyte hypertrophy (Bang et al., 2014), cardiac fibrosis (Bang et al., 2014; Yamaguchi et al., 2015), apoptosis (Barile et al., 2018; Qiao, Hu et al., 2019). Numerous pre-clinical researches exhibit the therapeutic potential of EVs in cardiovascular regeneration and protection (Lai et al., 2010; Giricz et al., 2014; Gallet et al., 2017). In conclusion, compared with cell-based therapy, EVs present the following advantages to CVDs therapy: 1) EVs are lack the self-replicating ability and have no tumorigenic potential (Laggner et al., 2020); 2) Constituent and function of EVs are relatively stable (Im et al., 2017); 3) EVs can cross biological barriers and reach the ischemic injury area easily (Kooijmans et al., 2016); 4) EVs can be easily modified and stored (Casado-Díaz et al., 2020); 5) EVs exhibit the same biological properties with their very source and can carry a variety of bioactive molecules to the recipient (Kim et al., 2018); 6) Obviate the need for transplantation of large numbers of cells. However, the potential of EVs is limited in several aspects: bioactivity, biodistribution, targeting, intracellular trafficking, and internalization. These limitations may be overcome by enhancing native EVs through pre- and/or post-isolation techniques before EVs-based therapeutics in clinically. In bioengineering approaches, researchers try to improve EVs’ bioactivity, biodistribution, delivery, targeting efficiency, and intracellular trafficking by modifying the surfaces of EVs *in vivo* (de Abreu et al., 2020). Therapies based on native and engineered EVs have been used to improve cardiac function in inflammation, cardiomyocyte death, fibrosis, and infarct size, and increased angiogenesis through transplantation (de Abreu et al., 2020).

In this review, we elaborated on the biophysical properties of EVs in the application of CVDs therapy. We also discuss the role of EVs in prognostic and diagnostic biomarkers in clinical. Particular attention will be paid to the bioengineered EVs which can favorably alter their bioactivity, targeting, internalization, and intracellular trafficking by modulating the native Evs’ surface.

### The Biophysical Properties of EVs

#### The EVs Size and Importance in Trafficking/Molecular Transport

**FIGURE 1:**
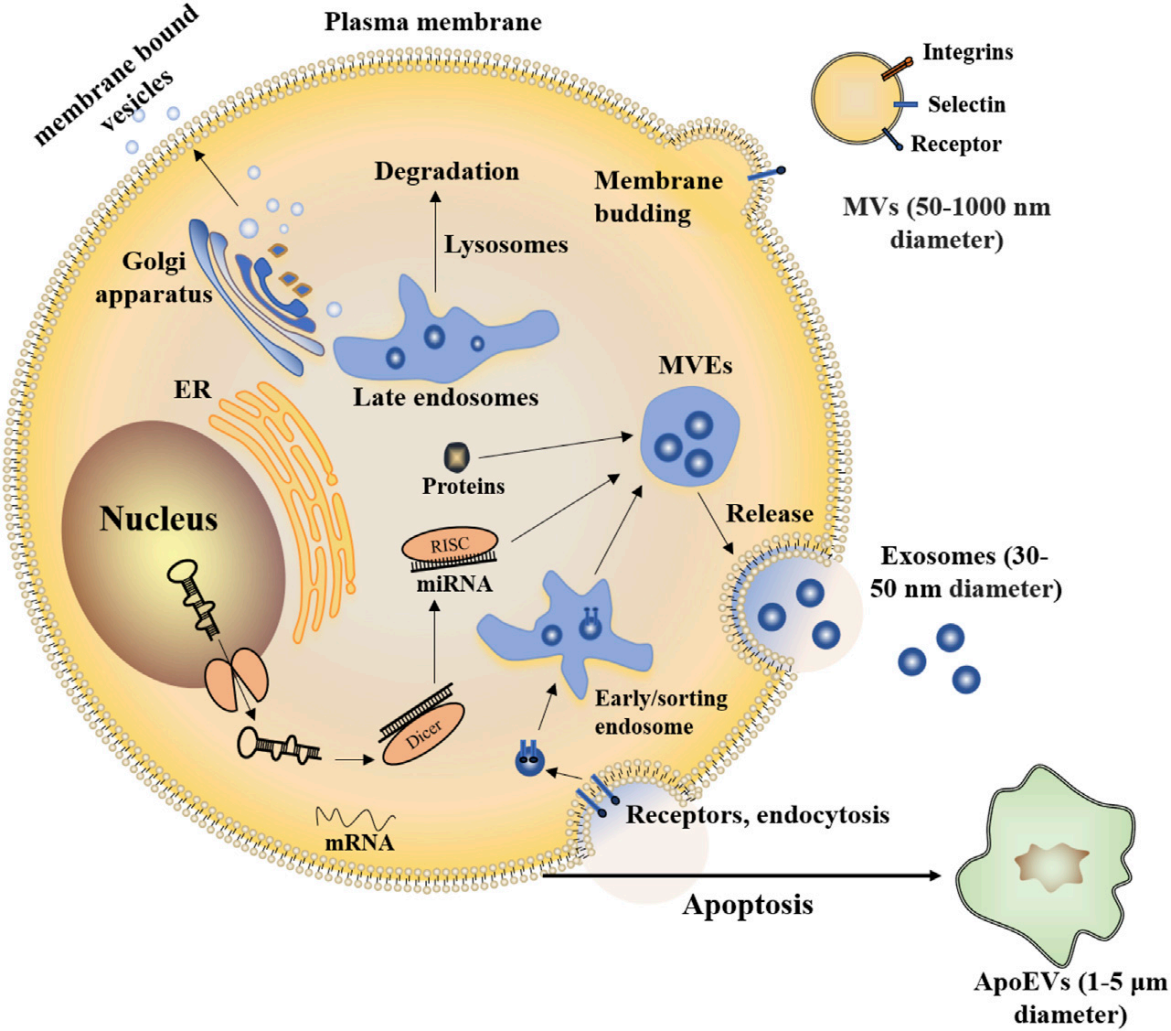
Exosomes and MVs are both released by healthy cells. Exosomes are nanometer-sized vesicles of endocytic origin that form by inward budding of the limiting membrane of MVEs. MVs bud from the cell surface. Part of the endomembrane system in the cytoplasm includes the endoplasmic reticulum (ER), Golgi apparatus, and lysosomes, it helps to package proteins inside the cell through membrane-bound vesicles. Vesicles also allow exchanging membrane components with a cell’s plasma membrane. Similar to healthy cells, apoptotic cells can also release EVs (termed apoptotic extracellular vesicles, ApoEVs).

The prevailing view on EVs’ classification depends on the diameter and origin. Several subtypes of EVs have been identified, such as exosomes, membrane vesicles, apoptotic bodies, and MVs (O'Brien et al., 2020). Exosomes are released from cells *via* the endolysosomal pathway. Exosomes are formed by inward budding of the limiting membrane of multivesicular endosomes (MVEs). The diameter of exosomes is 30–50 nm and MVs (also referred to as ectosomes, 50–1000 nm diameter), budded directly from the plasma membrane. The apoptotic bodies (1–5 μm diameter) are derived from the apoptotic cell membrane (Han et al., 2021) (Figure 1).

EVs size is crucial for the composition, tissue biodistribution efficiency, and intracellular trafficking in the application of CVDs therapy (Théry et al., 2006; Théry et al., 2018; Cabeza et al., 2020). For example, larger aggregates are more likely to be associated with membrane recycling or lysosomal degradation (Lim and Gleeson 2011). However, smaller vesicles (diameter <100 nm), were taken up *via* clathrin- or caveolae-mediated endocytosis (Costa Verdera et al., 2017). Therefore, smaller EVs may be more efficiently delivered into the cell. In the cardiac environment, especially for systemically administered EVs, the volume of EVs is critical relative to the successfully penetration into the heart tissue. So that EVs can be effectively absorbed by the relevant cell types (Liu et al., 2020).

#### EVs Potential and Interaction With Ligands Were Promoted Uptake by Target Cells

The surface potential of EVs is another important property. The EVs potential depends on the sugar composition of the plasma membrane (Akagi et al., 2014), which is rich in phosphate groups. In other words, the global negative charge is the norm for EVs. The changes in surface charge can be used to infer the stability of EVs in suspension. With the reduction of repulsive force, EVs accumulate mostly. The surface potential of EVs is the key to the interaction between EVs and many potential ligands, as well as their uptake by target cells (Ayala et al., 2013; de Abreu et al., 2020). In addition, more and more studies have shown that EVs play important roles in hemostasis and thrombosis due to the exposure to negatively charged procoagulant phospholipids (PPL) (Francula-Zaninovic and Nola 2018).

#### EVs Structure and Biological Content

For the structure of EVs, there is some structural similarity between cells and vesicles, both of which are lipid bilayer structure and negative potential. EVs are less susceptible to the penetration of small solutes due to their high cholesterol content (de Abreu et al., 2020). Benefiting from the external structure, EVs ensure the safe and efficient transmission from internal content to target cells. More interestingly, membrane composition differs from different types of EVs.

**FIGURE 2:**
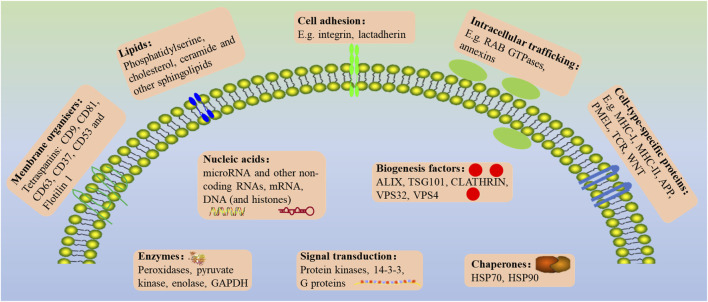
The general outlook of the EVs membrane composition and different molecular cargoes can markedly vary based on the parental cell and vesicle biogenesis.

The biological contents of EVs consist of various bioactive substances, including nucleic acids (DNA and RNA), proteins (biogenesis factors, enzymes), lipids, and metabolites (Jeppesen et al., 2019). The microRNA (miRNA), transfer RNAs (tRNAs), messenger RNA (mRNA) and fragmented mRNAs, long-stranded non-coding RNAs (lncRNAs), and circRNA are all found in EVs though the concentrations of RNA are relatively low (Li J et al., 2021). Proteins, such as Membrane surface markers (annexins and GTPases), lysosomal-associated membrane proteins 1 (LAMP1 and LAMP2), heat shock proteins (HSP 70 and HSP 90), tetraspanins proteins (CD9, CD63, CD37, CD53, CD81, and CD82), phospholipases, and other lipid-related proteins, are used to identify and isolate cell-type-specific EVs (Loyer et al., 2018). As the major part of EVs, RNAs and proteins don’t exist in the cytoplasm randomly (Valadi et al., 2007). Compared with lncRNAs and miRNA, circRNA is rarely studied, which is likely to become the next hot molecule for exosomes detection due to its unique stability, tissue specificity, timing, and disease specificity (Shi et al., 2020) (Figure 2). In addition to directly cell-cell contact or the transport of secreted molecules, EVs also participate in intercellular communication. By containing and transporting various bioactive molecules to target cells, EVs could affect biological behaviors and gene phenotypes through several molecular pathway’s regulation.

#### EVs-Content Release Within Target Cells

EVs have been proposed to transfer membrane encapsulated cargoes from donor to acceptor cells. However, the mechanism of EV-content release within acceptor cells remains debated. There is no consensus on the uptake mode of EVs, whether is receptor-dependent or not. High-resolution microscopy or new living cell reporter genes are needed for the research on EVs-content delivery within target cells (Sung et al., 2020), and reporter gene assay could also be used to measure the EVs membrane fusion efficiency during cargo delivery to receptors (Somiya and Kuroda 2021). Currently, studies have shown the way that EVs enter cells including receptor-mediated endocytosis, clathrin interaction, lipid raft interaction, phagocytosis, micropinocytosis, and possible directing fusion (Kalluri and LeBleu 2020). In addition, several pieces of research have shown that most EVs may not be absorbed by uninjured or chronically damaged heart tissues, but by non-cardiac cells (Yi et al., 2020; Kang et al., 2021).

#### EVs for CVDs Applications

##### Roles of EVs in the Prognostic and Diagnostic Biomarkers for CVDs

Currently, the treatment of CVDs includes traditional pharmacotherapy and surgery, which is costly and exhibits great side effects (Leong et al., 2017). The lack of biomarkers that limit the progress and treatment clarifies the pathogenesis of CVDs. Therefore, looking for new diagnostic, therapeutic, and prognostic approaches to prevent and treat CVDs is the major health priority. The biomarkers are used in clinical widely for the acute coronary syndrome (ACS) and HF including cardiac troponin, B-type natriuretic peptide, and so on. However, it is still very difficult to detect these biomarkers recently. For example, the use of highly sensitive methods to detect cardiac troponin I will accompany the loss of diagnostic specificity of acute myocardial infarction (AMI). Atrial fibrillation and renal insufficiency induced a decrease in diagnostic specificity in the diagnosis of acute HF. With the continuous development of molecular diagnostic concepts, liquid biopsy-based on EVs can reflect the dynamic changes of the disease effectively and become a future direction for molecular diagnostic research.

Studies suggested that circulating EVs may be used as biomarkers to predict and diagnose CVDs. In a prospective study of around 60 patients with AMI, the platelet P2Y12 antagonist Tegretol reduces mortality by down-regulation of the plasma EVs concentrations during AMI (Gasecka et al., 2020). In β-thalassemia patients with pulmonary arterial hypertension (PAH), the large Red Blood Cell-EVs, platelets, and medium platelet-derived EVs carrying phosphatidylserine (PS) were increased, compared with normal subjects (Manakeng et al., 2018). These phenomena suggest that the number of EVs may be a useful marker of disease stratification. Similarly, the contents of EVs could also be a useful marker for determining the severity of CVDs and clinical prognosis. A clinical trial around CVDs patients exhibited that the lncRNAs AC100865.1 (referred to as CoroMarker), as a diagnostic model from Fisher’s criteria could increase sensitivity significantly from 68.29% to 78.05%, while specificity decreased slightly from 91.89% to 86.49% in CVDs diagnosis (Yang et al., 2015). This evidence suggests that CoroMarker can be used as a stable, sensitive, and specific biomarker to determine the progression of CVDs in clinical. During the measurement of coronary circulation concentration gradients, it was found that miR-133a and miR-499 were enriched in cardiac myocytes in patients with troponin-positive acute coronary syndrome, which were released from the heart into the coronary circulation during myocardial injury, while vascular miR-126 was depleted (S., De et al., 2011). Increased expression of miR-199a in EVs but not plasma has been associated with major adverse cardiovascular events reduction in patients (Jansen et al., 2014). In addition, the level of miR-208a in serum exosomes was significantly higher in patients with ACS, compared to healthy individuals and the one-year survival group (Bi et al., 2015). It may provide an important point that miRNA may also act as biomarkers for CVDs prediction.

These studies illustrated the potential of both EVs and their contents can act as biomarkers in determining the occurrence, severity, and clinical prognosis of CVDs. Clarifying the relationship between the changes in EVs and CVDs will supply more evidence to support the clinical application of EVs. Additional biomarkers can help diagnose AMI quickly and specifically. The relationship between the components of EVs and the disease process is complex. Multiple biomarkers applied together can help reflect CVDs progression effectively, compared with single molecules (Han et al., 2021). Cardiomyocyte death and inflammatory stimulation can also promote fibrosis and induce coronary artery occlusion in ischemic heart disease, through the secretion of extracellular matrix (ECM) proteins (Prabhu and Frangogiannis 2016). It is reported that the levels of CD3^+^/CD45^+^ and SMA-α^+^ EVs increase in individuals with high cardiovascular risk (Niel et al., 2018). EVs-derived proteins can reflect the dynamic changes of CVDs specifically. Another study showed that the elevated level of cystatin C, serine protease inhibitors F2, and CD14 protein in plasma EVs is associated with the occurrence events of CVDs (Kanhai et al., 2013). In summary, the above studies illustrated that the correlation between EVs levels and CVDs status is close. Undoubtedly, EVs play important roles in the prognosis and diagnosis of CVDs. But further research on the specific relationship between EVs and CVDs is still needed.

#### Application of EVs in CVDs Therapy.

**FIGURE 3:**
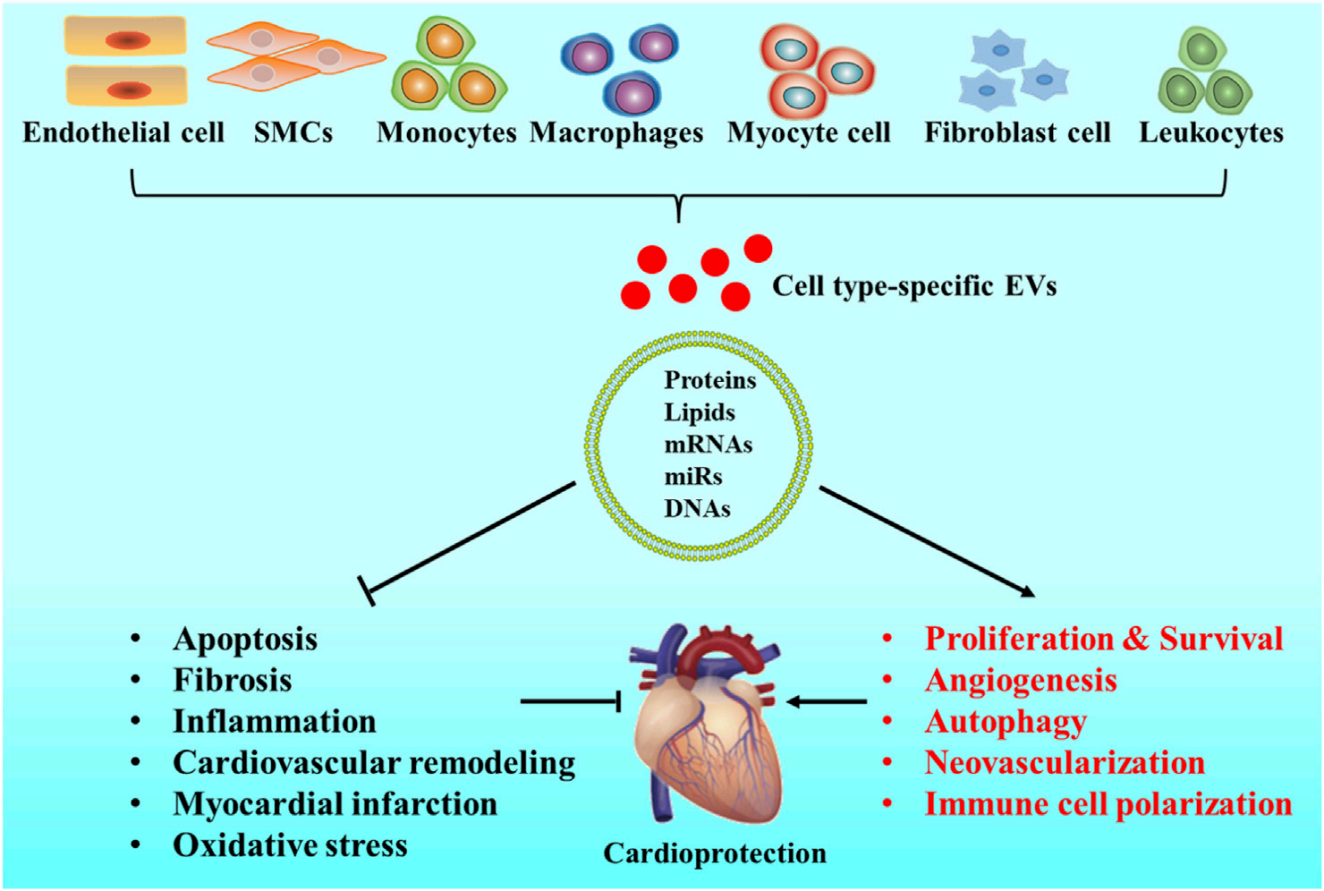
Origins and roles of EVs in CVDs. EVs can be released by cardiovascular system-related cells, such as cardiomyocytes, endothelial cells (ECs), fibroblasts, smooth muscle cells (SMCs), leukocytes, monocytes, and macrophages. EVs mimic the cardioprotective properties by stimulating cell proliferation, improving cardiac survival, activating cell autophagy, promoting angiogenesis, enhancing neovascularization, decreasing cell apoptosis, reducing tissue fibrosis, preventing inflammation, inhibiting cardiovascular remodeling, treating myocardial infarction, reducing oxidative stress levels, and affecting immune cell polarization.

EVs are a group of heterogeneous natural particles that can be used for CVDs therapy. More and more evidence highlights that EVs exhibit potential therapeutic function in CVDs (Sánchez-Alonso et al., 2018). Certain properties in these endogenous vesicles enable them to survive in the extracellular space, bypass biological barriers, and transport their biologically active molecular cargo to recipient cells (Nawaz and Fatima 2017; Kalluri and LeBleu 2020). The biological function of EVs depends on the state of donor cells and can vary during the different microenvironments (Genschmer et al., 2019). EVs containing miRNAs and proteins regulate multiple functions in target cells, including maintaining cardiovascular balance and health, inducing pathological changes in CVDs. Therefore, the fascinatingly complex features of EVs should also be taken into consideration in clinical applications (Han et al., 2021). EVs carried with miRNA-21 can effectively inhibit apoptosis and restore cardiac function *in vivo* and *in vitro* (Song et al., 2019). The therapeutic benefits of EVs in CVDs have also been confirmed by large animal models such as pigs and nonhuman primates (Li Q et al., 2021; Yao et al., 2021). The therapeutic effect of EVs has also been evaluated in several kinds of diseases through small animal models, including MI (Couto et al., 2017), hindlimb ischemia (Prabhu et al., 2017), and stroke (Tian et al., 2018). Studies have shown that EVs from different sources can trigger a variety of cardioprotective effects (Figure 3) (Benjamin et al., 2017; Jie et al., 2017; Fu et al., 2020). EVs isolated from the plasma of healthy volunteers can protect myocardium from ischemic reperfusion (I/R) injury or promote angiogenesis in the ischemic limb injury in animals (Vicencio et al., 2015; Aday et al., 2021). Increasing evidence suggests that the effects of EVs on target cells are mainly dependent on miRNAs and proteins transferred by EVs (Benjamin et al., 2017). Cardiomyocytes release EVs with high expression of miR-217, which act on fibroblasts and promote the proliferation of fibroblasts. These results indicate that miR-217 plays an important role in cardiac hypertrophy and dysfunction (Nie et al., 2018). Cardiomyocytes can promote cardiac fibroblast proliferation and myofibroblast differentiation by releasing EVs containing a high level of miR-208a (Yang et al., 2018). Related studies have also shown that EVs derived from platelets containing polyubiquitin, which can reduce platelet aggregation and inhibit the expression of CD36 through ubiquitination, thereby inhibiting the formation of atherosclerotic thrombosis (Srikanthan et al., 2014). EVs can act as a drug and ideal drug carrier in therapy for their benefit on circulation, immune rejection and cellular toxicity. As drug carrier, EVs exhibit potentials on protecting bioactive cargoes from degradation and higher transmission efficiency, compare with common liposomes (Barile and Vassalli 2017).

In consideration of that cardiac is lack of regenerative capacity following MI. Stem cell therapy has recently been applied to improve cardiac repairs in research. Early studies have found that stem cells, especially modified stem cells, show significant therapeutic potential in CVDs. During therapy, the differentiation degree can determine the efficacy of stem cells (Mangi et al., 2003; Kawamoto et al., 2006). Gnecchi’s group also found that the higher expression of Akt in mesenchymal stem cells (MSCs) the shorter recovery time cardiac function has, which means that modified stem cells are optimized for CVDs therapy (Gnecchi et al., 2006). Stem cells have a great potential for tissue regeneration and repair. However, stem cells have the ability of self-renew and proliferate indefinitely. The clinic application of stem cells is limited due to the teratoma risk (Nawaz et al., 2016). Recent findings elucidate exchange of genetic information utilizing persistent bidirectional communication mediated by EVs could regulate stemness, self-renewal, and differentiation of stem cells (Nawaz et al., 2016). Studies found that MSCs transplantation accelerated angiogenesis and improved cardiac repair after MI (Müller et al., 2018; Liang et al., 2021). Subsequently, the mechanism of MSCs mediated paracrine has been accepted and validated in exploring the principal mechanism of stem cells for CVDs therapy (Fan et al., 2020). It has emerged that the paracrine functions of MSCs could, at least in part, be mediated by EVs. EVs have significant potential as a novel alternative to CVDs. Studies have also found that native EVs used for CVDs could be derived from MSCs (Gollmann-Tepekylü et al., 2019), cardiac progenitor cells (CPCs), cardiosphere-derived cells (CDCs) (Couto et al., 2017), embryonic (ESCs), induced pluripotent stem cells (iPSCs) (Adamiak et al., 2018), dendritic (DCs) (Liu et al., 2016), and endothelial progenitor cells (EPCs). Compared to cell-based therapies, EVs may exhibit a superior safety profile such as a lower propensity to trigger innate and adaptive immune responses and the inability to form tumors directly (Rani et al., 2015). Moreover, the isolation of EVs from stem cells is potentially sustainable and reproducible. Compared with cells, EVs can be stored with high efficiency safely and easily (Ran et al., 2015).

MSC-derived EVs (MSC-EVs), derived from different origins such as bone marrow, adipose tissues, umbilical cord and heart, have exhibited comprehensive protection and reparation effects on cardiovascular (Racchetti and Meldolesi 2021). MSC-EVs can reduce cardiomyocyte apoptosis and cardiac fibrosis, but promote angiogenesis *via* the transfering bioactive miRNA, lncRNA, and protein cargos into targeted cells (Peng et al., 2020; Gca et al., 2021). CDC and CPC-derived EVs (CDC-EVs and CPC-EVs) have also been extensively used in MI or I/R injury (Barile et al., 2016; Romain et al., 2016). Studies had proved that CDC-EVs was safe and effective during repairing heart tissue damaged in HF (Raj and Mohsin 2017; Ibrahim et al., 2019). Other studies have shown that CDC-EVs can also reduce infiltration and inhibit cardiomyocyte apoptosis *via* transferring Y RNA fragment (EV-YF1) and miRNA-181 to the macrophages (Couto et al., 2017). Importantly, miR-147, miR-18, miR-133, miR-206, miR-10, miR-142, miR-146a were enriched in CDC-EVs and performed protective effects (Ibrahim et al., 2014). In addition, CPC-derived exosomes have also exhibited cardiac protection by reducing cell apoptosis and poor remodeling (Xiao et al., 2016). Another study found that human CPC-EVs reduced myocardial infarction by reducing cardiomyocyte death and promoting angiogenesis (Wu et al., 2020).

The iPSCs-derived EVs (iPSCs-EVs) also provide a cell-free system to avoid the risks associated with direct cell transplantation (Chandy et al., 2020). Regarding iPSC-EVs, miRNA is also an important functional component. As reported, miR-19, miR-20, miR-126, miR-130, and miR-17 derived from iPSCs exert a powerful effect on promoting angiogenesis, adjusting hypoxia, and oxidative stress. In addition, bioinformatics analyses showed that miRNA in iPSC-EVs can prove the cellular functional state to inhibit apoptosis through regulating Wnt, phosphatidylinositol-3 kinase/protein kinase B (PI3K-Akt), and mitogen-activated protein kinase (MAPK) pathways (Adamiak et al., 2017).

ESC-derived EVs can also augment cardiac function effectively in infarcted hearts through enhancing neovascularization, cardiomyocyte survival and proliferation, but inhibiting fibrosis in cardiac. This beneficial effect of ESC-derived EVs was linked to miR-294 was delivery from ESC to CPCs specifically, then increased survival, cell cycle progression, and proliferation (Adamiak and Sahoo 2018). The study showed that human CD34^+^-positive EPCs exhibited the potential on CVDs therapy (Sahoo et al., 2011; Sahoo and Losordo 2014) and promote proangiogenic paracrine activity in ischemic limb tissues (Prabhu et al., 2017). Further studies shown that EPCs-derived EVs (EPCs-EVs) could increase the formation of new blood vessels and improved left ventricular function in patients with MI (Yue et al., 2020). In addition, EPCs-EVs could also enhance blood vessel formation by promoting the transformation of fibroblasts into endothelial cells (Huang, et al., 2021; Ke, et al., 2021). DC-derived exosomes were involved in activation ECs by TNF-α and NF-kB signaling pathways in human umbilical vein endothelial cells (Jadli et al., 2021).

In conclusion, EVs were identified as the major component of stem cell secretome responsible for the observed increase in cardiac function. The contents of EVs play key roles in CVDs therapy, and their effects can be summarized as follows: 1) Inhibit apoptotic; 2) Reduction of oxidative stress; 3) Reduction of fibrosis; 4) Regulation of autophagy; 5) Reduction of inflammatory response; 6) promotion of angiogenesis; 7) Stabilization of mitochondrial membrane potential. As reported, the stem cell-derived EVs in CVDs therapy included MSC-EVs, CDC-EVs, iPSC-EVs and DC-EVs. They can help to carry different microRNAs to cardiac develop their therapy function (Table 1).

**TABLE 1:** Origins and therapeutic application of stem cell-derived EVs in CVDs.

Classification	Origins	Functional Contents	Functions	References
MSC-EVs	Mesenchymal stem cells	miR-19	(a) reduce cardiomyocyte apoptosis	(Saad et al., 2016; Wen et al., 2017; Moghaddam et al., 2019; Pan et al., 2020)
		miR-21	(b) reduce cardiac fibrosis	
		miR-210	(c) promote angiogenesis	
		Growth factor-D	(d) stabilize mitochondrial membrane potential	
CDC-EVs	Cardiosphere-derived cells	EV-YF1 miRNA-181	(a) reduce oxidative stress	(Cheng et al., 2014; Rustagi et al., 2015; Gabbia et al., 2021)
			(b) promote angiogenesis	
			(c) reduce cardiac fibrosis	
iPSC-EVs	Induced pluripotent stem cells	miR-21	(a) adjust hypoxia and reduce oxidative stress	(Treguer et al., 2012; Li et al., 2018; Moghaddam et al., 2019; Atum et al., 2021)
		miR-24	(b) promote angiogenesis	
		miR-294		
		miR-19		
DC-EVs	Dendritic	miR-494	(a) reduce inflammatory response	(Rana et al., 2013; Mao et al., 2015; Espinosa-Diez et al., 2018)
			(b) promote angiogenesis	

The potential of EVs is limited in multiple factors, including bioactivity, biodistribution, targeting, intracellular trafficking, and internalization. Variations and limitations in EVs isolation techniques, basic characterization, and precise dosing regimens can affect study results. The expected biological effects of EVs are mostly produced from internalization of recipient cells through endocytosis pathways (Mulcahy et al., 2014). Numerous studies have found that intravenously administered EVs are rapidly cleared by macrophages and accumulated in mononuclear phagocyte system (MPS) organs such as the liver, spleen, and lung (Chen, Wang et al., 2021). Compared with intracoronary or intravenous administration, intramyocardial administration of EVs can increase the lifetime of EVs in heart. Results showed that intramyocardial delivery of EVs can improve left ventricular ejection fraction and reduce the infarct size, regardless of its source (de Abreu, Fernandes et al., 2020). However, intramyocardial delivery of EVs is complex in a clinical catheterization (Gallet et al., 2017). Targeted technology can increase the accumulation and decrease the application dose of EVs in the cardiovascular system. The strategy of using specific biomolecules to increase the content of EVs may be the key to its successful clinical application. Currently, three strategies for targeted delivery of therapeutic EVs to the heart have been reported: 1) encapsulation of EVs in hydrogels, 2) genetic engineering of EVs, and 3) two-step EV delivery. In summary, three strategies can shorten the time that EVs take to reach their therapeutic targets and significantly reduce off-target effects, thereby improve therapeutic efficacy (Chen et al., 2021). To improve the efficacy of native EVs in CVDs, researchers have also developed technologies to improve the biological activity and stability of EVs in the cardiovascular system. The bioengineered EVs can be obtained by modulating the source of cells, genetics, metabolic engineering, and chemical or physiological methods (Huang et al., 2019; Hao et al., 2020). Cardiac homing peptide (CHP) was used to conjugate with EVs with a special linker. Modified EVs exhibited a longer lifetime in myocardial tissue as well as better functional status in the heart after injecting intravenously (Wen et al., 2019). The protein or peptide modified lipid is physically incorporated into the EVs membrane, or the linker is chemically coupled to the functional groups on the surface of the EVs. Compared with traditional bio-combination technology, the modified lipid is fast, more selective, and efficient. Chemical structure modification can change Evs’ surface and targeted epitopes’ density effectively, regardless of the source of the cell. In addition, the chemical method can be carried out during the purification process of EVs. Therefore, it is more suitable for clinical application (de Abreu et al., 2020). In conclusion, the modified EVs were enriched in therapeutically relevant compounds, and decorated with surface epitopes that improved their cardiac targeting and pharmacokinetics. Therapies based in modulated EVs exhibits improvement on cardiac function through decreasing in inflammation, cardiomyocyte death, fibrosis and infarct size, as well as increasing angiogenesis.

## Perspectives and Challenges

**FIGURE 4:**
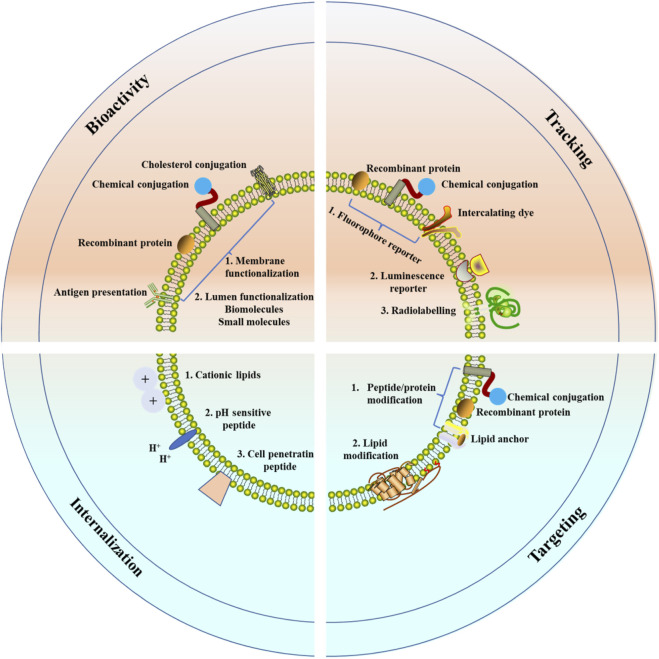
Modulation of EVs for CVDs therapy. Several strategies have been used to overcome the limitations of native EVs. To enhance the therapeutic potential of EVs, the membrane and the lumen have been functionalized. To track *in vivo* EVs, fluorophores, luminescence reporters, or radiotracers have been used to label formulations. To improve the targeting efficacy of EVs, exogenous peptides, proteins or lipids have been modified. To enhance EV internalization and endolysosomal escape, the vesicles have been modified with cationic lipids, pH-sensitive peptides, and cell-penetrating peptides.

The observational or interventional clinical trials involving EVs grow continually in cancer therapy (Eitan et al., 2017; Kontopoulou et al., 2020). Several clinical trials in the treatment of CVDs or acute ischemic stroke have exhibited that, no major adverse events were observed during EVs clinical application (Sciences 2021; Xinhua Hospital 2021). The clinical transformation of EVs as potential therapies still faces some challenges. Firstly, further technologies are needed to overcome the challenges in isolation, purification, characterization, and long-term storage of EVs, which are crucial for the quantification of EVs (Hao, Song et al., 2021). EVs are heterogeneous, and there are no methods or specific markers could help to distinguish exosomes, small MVs, or exosome subgroups, which limit the application of EVs in therapy. Secondly, after entering the circulation system, EVs must be avoided digestion in the liver, lung, kidney, or other organs and immune cells (Herrmann et al., 2021), as well as other targeting cells. These systemic treatments may be limited due to off-target effect. Finally, the application of EVs in the cardiovascular area also requires standardized sources. EVs can be harvested from autologous or exogenous sources. Their immunocompatibility makes it impossible to be on-demand production, and it is more difficult to standardize their production (de Abreu et al., 2020). Therapy with bioengineered EVs will be a promising, cell-independent, durable and customizable way to improve the progrosis factors of CVDs patients (Figure 4).

## Conclusion

Over the past decade, significant progress has been made to understand the biological characteristics of EVs, that helps to enhance EVs’ role as CVDs drug delivery vehicles, acted in diagnosis, prognosis, therapy, and clinical transformation. The severity of CVDs and their progression can be reflected by detecting changes in the circulating levels and biological composition of EVs, or by detecting altered circulating levels of EVs containing specific surface molecules and contents. Although the specific relationship between circulating levels of EVs and CVDs is known little currently, EVs are still used as biomarkers in determining cardiovascular function and disease progression. To study the role of EVs in the occurrence and progression of CVDs, more analysis of the relationship between EVs and the clinicopathological features of CVDs should be conducted, and further exploration of their targeted therapy options is needed. These will help treating CVDs, prevent the further deterioration of CVDs, and promote the development of EVs in the clinical setting. Moreover, EVs are using in regenerative medicine currently, which indicates that EVs exhibit great potential in CVDs therapy. Ultimately, EVs are robust and promising approaches to improve outcomes for patients with CVDs.
